# Novel heterozygous *ASH1L* nonsense variant involved in mild intellectual disability

**DOI:** 10.3389/fneur.2025.1524532

**Published:** 2025-01-20

**Authors:** Baoqiong Liao, Wuming Xie, Shuwen He

**Affiliations:** ^1^Ganzhou Maternal and Child Health Hospital, Ganzhou, Jiangxi, China; ^2^Medical Genetic Diagnosis and Therapy Center of Fujian Maternity and Child Health Hospital College of Clinical Medicine for Obstetrics & Gynecology and Pediatrics, Fuzhou, Fujian, China; ^3^Ganzhou People^’^s Hospital, Ganzhou, Jiangxi, China; ^4^Department of Chemistry and Molecular Biology, Gothenburg University, Gothenburg, Sweden

**Keywords:** *ASH1L*, intellectual disability, onsense mutation, WES - whole-exome sequencing, neuroscience

## Abstract

Mutations in *ASH1L* have been associated with a range of phenotypes, including intellectual disability (ID), autism spectrum disorder (ASD), attention deficit hyperactivity disorder (ADHD), seizures, as well as differences in skeletal, muscular, and sleep functions. In this study, we describe a patient diagnosed with mild ID, and whole-exome sequencing (WES) of the family identified a novel heterozygous nonsense variant, NM_018489.2: c.2479A > T (p.Lys827*), located in exon 3 of *ASH1L*, which was predicted to be pathogenic. The nonsense variant in the mild ID patient may disrupt *ASH1L* function by destabilizing its spatial conformation, leading to decreased activity of the catalytic H3K36 methylation, thereby affecting neurological function. A review of reported *ASH1L* nonsense mutations to explore genotype–phenotype correlations suggested that these variants typically result in a loss of function. Our findings contribute to understanding the neurodevelopmental pathogenesis of mild ID in patients with the *ASH1L* nonsense variant mutation.

## Introduction

Intellectual disability (ID) includes a variety of developmental disorders defined by limitations in cognitive abilities and adaptive behavior according to the diagnostic and statistical manual of mental disorders, 5th Edition (1). An intelligence quotient (IQ) under 70 implies a deficit in intellectual functioning, which in combination with adaptive functioning determines further classification as severe, or profound, moderate and mild. ID can manifest in varying degrees of severity and is often accompanied by challenges in learning, communication, and daily life skills. The onset of ID typically occurs before the age of 18 and affects approximately 1–3% of the global population (2–4). ID is primarily limited to individuals over 5 years old, while global developmental delay (DD) is used for children 5 years old or younger (5, 6).

ID may arise from both genetic and non-genetic factors. Non-genetic causes include nutritional deficiencies, exposure to toxic substances, maternal infections during pregnancy, hypoxic–ischemic events, brain radiation, encephalitis and traumatic brain injuries (7). However, a significant proportion of ID cases are attributed to genetic causes, such as chromosomal abnormalities including trisomies, deletions, and duplications (8, 9). Genetic syndromes associated with ID frequently present with additional clinical symptoms, including motor, psychiatric, and sensory impairments (10). These co-occurring conditions can make diagnosis more difficult and may hinder the correct identification. The children with congenital hearing loss may face developmental delays that resemble conditions like autism (11). Furthermore, sensory impairments like hearing loss may be mistaken for behavioral issues (12), complicating both diagnosis and treatment. Advances in genomic technologies, including array-comparative genomic hybridization (Array-CGH) and WES, have enabled the identification of many genetic variants linked to ID (13, 14). The discovery of these genes reveals its underlying causes and expands our understanding of the disease.

Among these genes, *ASH1L* (Absent, small, or homeotic1-like) has garnered significant attention due to its critical role in brain development. *ASH1L* (also referred to as *KMT2H*, *ASH1*-like, *ASH1L1*, *ASH1*, or *huASH1*) is a enzyme classified as a histone-lysine N-methyltransferase and encoded by the *ASH1L* gene which is located at chromosomal band 1q22 (15). As a member of the trithorax group family, *ASH1L* facilitates the methylation of specific histone lysine residues and lays a key role in regulating transcription and chromatin remodeling (16, 17). Mutations or loss of function in *ASH1L* were linked to various developmental disorders, including ID and ASD. These mutations typically result in a nonfunctional enzyme, disrupting histone methylation and altering gene expression, which in turn affects brain development. First described *ASH1L* variant in patients in clinical study involving three patients with ID or ASD due to *de novo ASH1L* missense variants (18–20). In addition, *ASH1L* mutation have also been linked to seizures, which broadens the diversity of both genetic and clinical features seen in *ASH1L*-related neurodevelopmental disorders (21). *ASH1L* mutations result in altered methyltransferase enzyme activity and changes in neuronal morphology, leading to cognitive impairments and disruption of *ASH1L*’s regulatory functions (22). Mutations within the *ASH1L* PHD-BAH domain may disrupt interaction with methyltransferase enzyme *in vitro*, potentially compromising chromatin remodeling (23). Mouse models with *ASH1L* exon 4 deletion resulting in a premature stop codon (p.V1693Afs*2) exhibit abnormal cortical neuron differentiation and craniofacial abnormalities, providing insights into the molecular mechanisms underlying *ASH1L*-associated neurodevelopmental disorders (24). While previous reports have identified several heterozygous loss-of-function (LOF) variants in *ASH1L*, such as nonsense, frameshift, and deletions, the phenotypic spectrum remains incompletely understood. In particular, the relationship between these mutations and the severity or specific features of the associated disorders is still being explored.

To address this gap, our study specifically aims to clarify the genotype–phenotype relationship of *ASH1L* variants, refine diagnostic criteria for *ASH1L*-associated neurodevelopmental disorders, and inform future research into targeted therapeutic strategies. Here, we identified a novel heterozygous nonsense variant of *ASH1L*, NM_018489.2: c.2479A > T (p.Lys827*), in a patient diagnosed with mild ID. This variant adds to the growing body of evidence supporting *ASH1L*’s role in neurodevelopmental disorders and expands the known phenotypic spectrum of this condition. Our findings emphasize the importance of larger-scale studies to further characterize the clinical impact of *ASH1L* nonsense variants and highlight the need for more comprehensive neurological evaluations.

## Materials and methods

### Subjects

The research informed consent was undersigned by the patient and parents. This study was carried out in accordance the ethics requirement. The study involved the proband and both parents. Family members were selected based on their relationship to the proband and symptoms of intellectual disability. The clinical assessment of Wechsler Adult Intelligence Scale (WAIS) was conducted prior to genetic testing to ensure thorough phenotypic characterization. The proband in our research is a 23-year-old female with a novel variant (c.2479A > T, p.Lys827*; NM_018489.3). She is the only child born to a non-consanguineous couple, and her prenatal, labor, and postnatal medical history are entirely normal, with no noted abnormalities. The proband has a score of 65 on the WAIS assessment, indicating borderline cognitive functioning. Additionally, it is reported that the proband has mild ID with poor learning abilities and poor memory. She can manage her daily life independently but shows slightly delayed responses. Her mother exhibits similar symptoms. Family history is important for the characteristics of ID on the mother’s side. The proband has no characteristics reported in other probands, for example, seizures. The proband is found to be non-dysmorphic. The results of Multiplex Ligation-dependent Probe Amplification (MLPA) test for the proband did not reveal any large segment variants within the detection range of the P070 kit. It is noteworthy that following clinical testing, this proband participated in a research study that uncovered four variants of unknown significance (VUS). The first is a missense variant in ACTL6B, which, according to OMIM, is associated with developmental and epileptic encephalopathies (OMIM#618468), and intellectual developmental disability with severe speech and walking deficits (OMIM# 618470). Another variant is a missense mutation in COQ8A, which has been linked to primary coenzyme Q10 deficiency (OMIM# 612016). The third is also a missense variant in DPP6 related to the intellectual developmental disabilities (OMIM# 616311). The last is an in-frame variant in SETD1B, which is associated with intellectual developmental disabilities accompanied by seizures and speech delays (OMIM# 619000).

### Genomic DNA preparation

Whole blood was collected in EDTA anticoagulant tubes (4 mL from the proband, 2 mL from parents and family members). Samples were processed and stored at −80°C to preserve DNA integrity during transportation to Kangxu Diagnostics (Beijing, China). Genomic DNA was extracted using the Qiagen FlexiGene DNA Kit, with quality assessed by Qubit 2.0 and NanoDrop 2000. We ensured ≥1.5 μg of DNA with a concentration of 50–100 ng/μL and OD 260/280 ratio of 1.8–2.0. DNA was fragmented into 180–280 bp segments, followed by end repair, A-tailing, adapter ligation, and exon capture using Agilent SureSelect Human ALL Exon V6 probes. Sequencing was performed on the T7 platform with 100× depth and PE150 reads. Libraries were validated using Qubit 2.0 and Agilent 2,100, with a minimum library concentration of 3 nM before sequencing.

### Genetic analysis and ACMG integration

The Ethics Committee of the Ganzhou Maternal and Child Health Hospital approved this research. Single-person WES was performed by Kangxu Diagnostics (Beijing, China). Given the proband’s complex phenotype, WES provided a more comprehensive genetic assessment, enhancing the likelihood of discovering relevant variants beyond well-characterized gene sets.Variants screening were based on clinical phenotypes of the affected subjects. Variants were filtered using biological information prediction tools (Polyphen2 Mutation Taster, SIFT and Splice Al), population database (ExAC, 1,000 Genome, dbSNP) and disease database (Clinvar., HGMD, OMIM). Thresholds for pathogenicity were determined based on ACMG guidelines, ensuring a standardized and reliable classification of variants. The ACMG guidelines were systematically applied to classify the pathogenicity of identified variants, incorporating multiple lines of evidence into a weighted framework: PVS1 (Pathogenic Very Strong): The novel variant identified in the ASH1L gene (c.2479A > T, p.Lys827Ter) results in a premature stop codon, predicted to lead to nonsense-mediated decay (NMD) and loss-of-function (LoF). This aligns with the established pathogenic mechanism of LoF for ASH1L-related disorders. PM2_Supporting (Pathogenic Moderate Supporting): The variant is absent in population databases, including ExAC and gnomAD, indicating it is a rare mutation. Following ACMG guidelines, the combined evidence supports classification of the ASH1L variant as “likely pathogenic.” The variant was confirmed by Sanger sequencing in the proband and family members, further validating its authenticity.

### Genotype–phenotype correlation

A review was conducted of all *ASH1L* truncating mutations (NM_018489.2) reported in HGMD through November 2024,^1^ with a summary of these mutations and their associated disease descriptions provided in Table 1. Truncating variants including frameshift and nonsense mutations cause significant protein malformations and typically lead to complete loss of function and haploinsufficiency.

**Table 1:** Nonsense mutations described in the *ASH1L* gene, with their nucleotide position and associated phenotype.

Mutation	HGMD access ID	Mutation type	Disease	PMID	Inheritance
160C > T	CM2316043	Arg54Term	Neurodevelopmental psychiatric disorder	36,475,376	Not determined
1348C > T	CM2234398	Gln450Term	Autism spectrum disorder	35,982,160	*De novo*
1420A > T	CM217232	Lys474Term	Neurodevelopmental disorder	33,860,439	*De novo*
1450C > T	CM217231	Arg484Term	Neurodevelopmental disorder	33,860,439	*De novo*
6427G > T	CM1617300	Glu2143Term	Autism spectrum disorder	27,824,329	*De novo*
6826C > T	CM222749	Arg2276Term	Intellectual disability and autism	33,879,512	Not determined
7189C > T	CM1513932	Arg2397Term	Autism spectrum disorder	26,325,558	*De novo*
7252C > T	CM2059558	Arg2418Term	Developmental disorder	33,057,194	*De novo*
7261C > T	CM180259	Arg2421Term	Intellectual disability/developmental delay	29,276,005	*De novo*
7603C > T	CM2234397	Arg2535Term	Autism spectrum disorder	35,982,160	*De novo*
8071C > T	CM2210986	Arg2691Term	Mental retardation, autosomal dominant	35,599,849	*De novo*
8731C > T	CM2316046	Arg2911Term	Neurodevelopmental psychiatric disorder	36,475,376	*De novo*
8887C > T	CM177684	Arg2963Term	Autism spectrum disorder	28,263,302	*De novo*

### Protein structure prediction

The protein sequence of *ASH1L* consisting of 2,965 amino acid residues was downloaded from uniprot web.^2^ By using the AlphaFold web server,^3^ the wild-type and mutant-type (*ASH1L*: c.2479A > T) 3D structure of the *ASH1L* protein could be predicted (25). Based on pLDDTs prediction scores, we selected the most reliable model for our studies, with higher scores indicating greater confidence in the structure. The prediction models were edited and visualized through PyMOL program.^4^

### Protein–protein interaction network and functional annotation analysis

The PPI network associated with *ASH1L* was constructed using STRING.^5^ The target genes in PPI network downloaded from STRING were used for reactome pathway enrichment analysis. The expression data was from the GTEX public database^6^ and was used to analyze the expression of *ASH1L* in different isoform and exon.

## Results

### Identification of the novel heterozygous *ASH1L* nonsense variant

**Figure 1:**
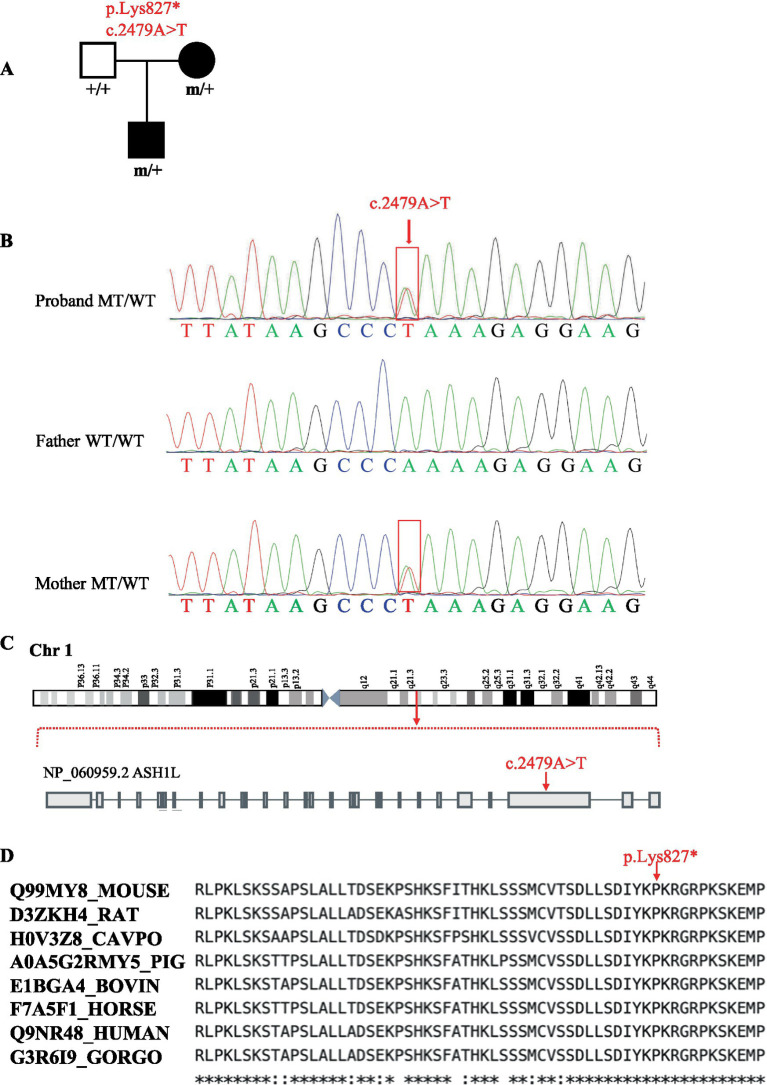
A novo variant of ASH1L were identified in the patients. **(A)** The pedigrees and genotypes of the families are presented, with probands having undergone whole-exome sequencing (WES). Filled symbols represent affected individuals. **(B)** Sanger sequencing chromatograms display the ASH1L variant identified in the families. **(C)** Localization of the ASH1L: c.2479A > T variant found in the study. **(D)** Amino acid conservation of the novel variant p.Lys827* in different species.

The patient’s whole-exome sequencing (WES) uncovered a novel heterozygous mutation (c.2479A > T/p.Lys827*) in the *ASH1L* gene. Cosegregation analysis revealed that the variant was passed down from the mother (Figure 1A). Sanger sequencing verified the presence or absence of this mutation in the family members (Figure 1B). Moreover, this variant have not been reported in any public population databases, including the 1,000 Genomes Project and GnomAD. The *ASH1L* c.2479A > T mutation is located on chromosome 1, specifically in exon 18 of the *ASH1L* gene. This mutation results in the substitution of adenine (A) with thymine (T) at nucleotide position 2,479, leading to the introduction of a premature stop codon at the protein level (p.Lys827*), which truncates the resulting protein (Figure 1C). The variant was conserved among species, including humans, rat, mice, cavpo, pig, bovine, and horse (Figure 1D). According to the American College of Medical Genetics and Genomics (ACMG) guidelines for interpreting sequence variants, this mutation was classified as pathogenic, based on two key factors: (1) it is a predicted loss-of-function (LOF) variant in a gene where LOF is a known cause of disease. (2) No additional point mutations or copy number variants (CNVs) in candidate genes, including those linked to epilepsy, were detected under models of autosomal recessive.

### Mutations in *ASH1L* carried by patient with ID affect the molecular structure

**Figure 2:**
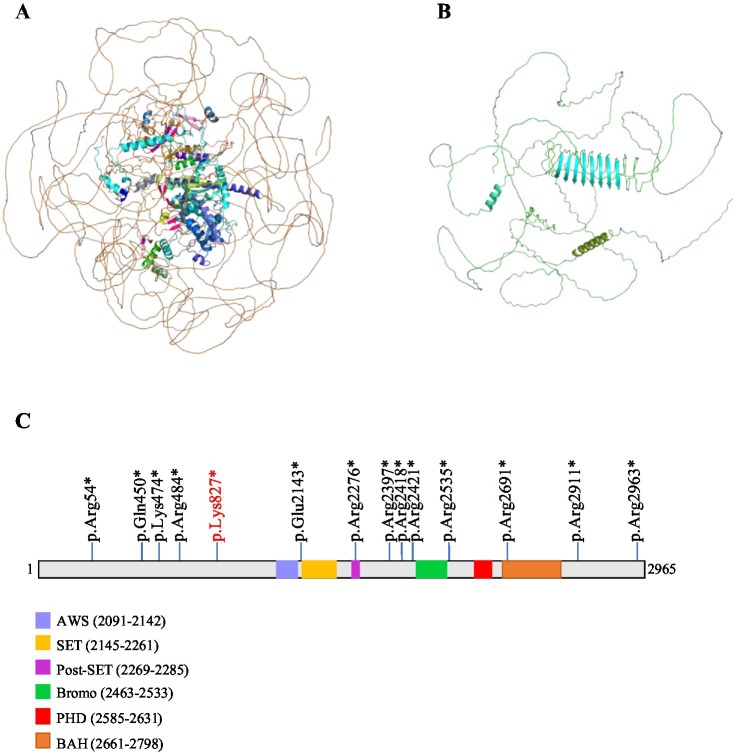
Potential impact of the c.2479A > T mutation on *ASH1L* protein structure. The wild-type structure of the *ASH1L* protein in **(A)** and the mutant protein structure shown in **(B)** contained a truncated *ASH1L* protein that contains 827 of the 2,695 amino acids of the mature protein. **(C)** Schematic diagram of ASH1L protein including five functional domains (AWS, SET, Post-SET, Bromo, PHD, BAH) are shown. All truncating mutations are indicated by sticks.

In order to analyze the effect of the novel variant NM_018489: c.2479A > T/p.Lys827*, on the structural integrity of the *ASH1L* protein, we employed the AlphaFold tool to predict potential changes in the protein’s structure. The NM_018489: c.2479A > T variant in *ASH1L* induces a frameshift mutation, resulting in a truncated *ASH1L* protein containing only 827 amino acids out of the 2,695 present in the full-length mature protein (Figures 2A,B). *ASH1L* is a multidomain protein composed of a long, unannotated N-terminus, a catalytic SET domain and three C-terminal histone binding domains: bromodomain (BRD), plant homeodomain (PHD) and bromo-associated homology (BAH) (26). The SET domain is responsible for histone methyltransferase (HMT) activity (16). The BRD domain of *ASH1L* targets acetylated chromatin to facilitating gene regulation. The BAH domain stabilizes *ASH1L*’s chromatin association and mediates interactions with other regulatory proteins (27). A crucial role of PHD domain is identifying histone modifications at specific sites and facilitating the recruitment of regulatory proteins (28). More and more research has reported that *ASH1L* mutations are associated with various neurodevelopmental disorders, including ID, ASD, and microcephaly (MCA) (Figure 2C) (19, 29, 30). The truncation caused by this variant results in the absence of key functional regions of *ASH1L*. Without these domains, the truncated *ASH1L* protein is likely unable to perform its typical biochemical functions. This alteration is expected to affect the stability of *ASH1L*, disrupt its enzymatic ability, and impede its role in chromatin remodeling, ultimately leading to disease pathology.

### Functional effect of the heterozygous *ASH1L* nonsense variant

**Figure 3:**
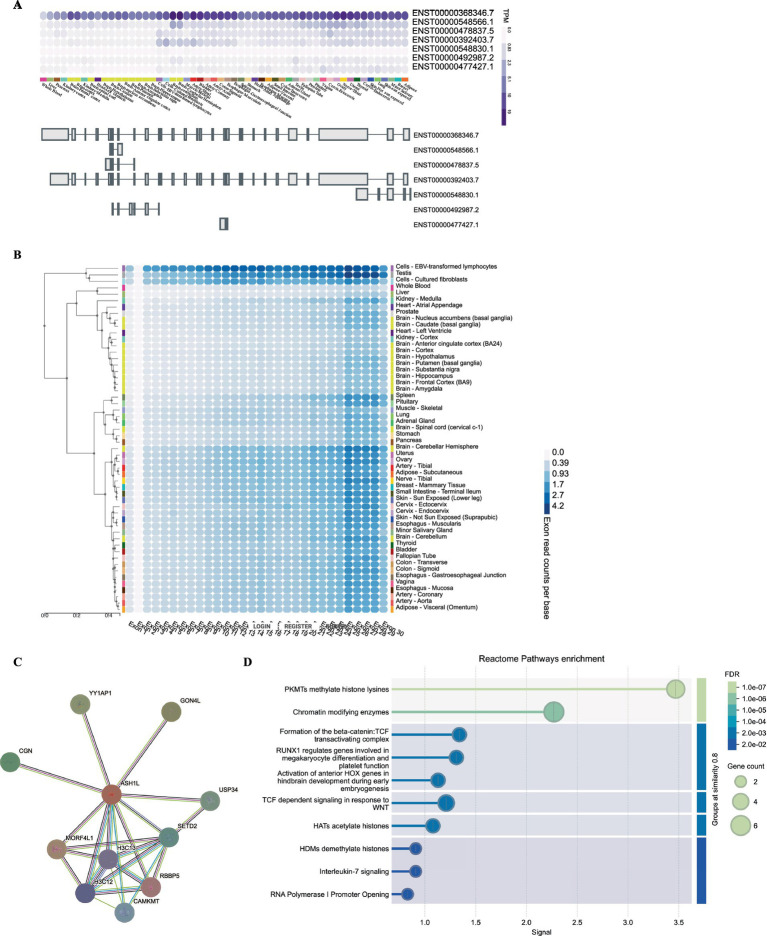
Gene expression and pathway analysis analysis of *ASH1L*. **(A)** GTEx data demonstrate that only a single, full-length *ASHIL* isoform (ENST00000368346.7) is expressed at an appreciable level in any adult human tissue represented. **(B)** The exon expression of *ASH1L* was obstained from GTEx data. **(C)** Bulk tissue gene expression for ASH1L. **(D)** The PPI network analysis of interacting genes with *ASH1L* and reactome pathway enrichment were downloaded from STRING.

To identify gene-level characteristics that could support the biological basis for such a cluster, we began by searching the GTEx (Genotype-Tissue Expression) (see text footnote 6) and discovered that the full-length, canonical transcript is the only isoform expressed at an significant level in any adult human tissue (Figure 3A). In further support of this conclusion, exon-normalized expression was uniformly distributed across all exons in each tissue analyzed from the GTEx dataset (Figure 3B). Next, we investigated whether the existence of *ASH1L* have broader functional genomic effects. We then conducted a PPI network analysis using STRING to investigate potential genes that interact with *ASH1L*. As shown in Figure 3C, the PPI network consisted of 11 nodes. The top three proteins with higher degrees are *MORF4L1*, *MORF4L1,* and *YY1AP1*. The GO enrichment analysis (Figure 3D) showed that these genes were enriched in regulating methylate histone lysines, chromatin modifying enzymes and TGF dependent signaling in response to WNT. This suggests a multifaceted role for *ASH1L* in regulating chromatin structure and signal transduction processes.

## Discussion

Mild ID is a chronic neurodevelopmental disorder characterized by limitations in cognitive functioning and adaptive behaviors, with an increasing incidence observed in recent years (31). However, current drug treatments for patients with mild intellectual disabilities are often unsatisfactory and seriously affect their physical and mental health, particularly among youth. The primary interventions for managing mild ID include a variety of therapeutic approaches, such as educational support and behavioral therapies (32). Despite the advancements made in treatment strategies, the development of effective therapies for mild ID individuals remain a critical challenge. Therefore, improving our understanding of its pathogenesis would contribute to the development of new treatment in clinical.

The present study advances our understanding of *ASH1L*’s involvement in neurodevelopmental disorders by identifying a novel heterozygous nonsense variant, NM_018489.2: c.2479A > T (p.Lys827*), in a patient with mild ID. This discovery is aligned with the *ASH1L*’s critical role in chromatin remodeling and gene transcription within neural development pathways (33, 34). Given *ASH1L*’s role as a histone-lysine N-methyltransferase, this mutation likely disrupts crucial epigenetic processes necessary for normative brain development. This disruption broadens the phenotypic spectrum associated with *ASH1L* mutations and strengthens the link between *ASH1L* loss-of-function variants and a range of neurodevelopmental outcomes, including ID and ASD. It has been highlighted *ASH1L* play an important role in epigenetic regulation, particularly through its function in catalyzing the methylation of histone H3 at lysine 4 (H3K4) (35). H3K4me3 promotes gene activation when NURF complexes are present. It maintains chromatin in an active “on” state through the plant homeodomain (PHD) domain. This allows transcription factors to access DNA within the chromatin (36). *ASH1L* regulates the expression of essential developmental genes by antagonizing polycomb- mediated gene silencing. This action finally limits the accessibility of target genes.

As seen in other neurodevelopmental disorders, H3K4 dysregulation can lead to varied neurobehavioral and cognitive phenotypes depending on the specific mutation and affected neural pathways (37–39). Moreover, previous research has established a phenotypic range in *ASH1L*-associated conditions with pathogenic variants linked to severe and mild cognitive impairments, language deficits, and neurobehavioral disturbances (40, 41). The presence of a novel nonsense variant, p.Lys827*, adds to this evidence, emphasizing the variability in clinical outcomes associated with *ASH1L* mutations. Mouse models deficient in *ASH1L* have displayed abnormal cortical neuron differentiation and craniofacial abnormalities, supporting a strong genotype–phenotype correlation and mirroring human neurodevelopmental features associated with *ASH1L* loss-of-function (42, 43). However, most human studies to date have focused on more severe clinical cases of *ASH1L*-associated ID. This makes our discovery of a nonsense variant in a mildly affected individual particularly relevant for expanding the clinical profile of *ASH1L*-related neurodevelopmental disorders.

Importantly, our findings in nonsense mutation demonstrates WES’s value in identifying rare variants and establishes *ASH1L* as a key candidate for further study in mild ID. A limitation of our study was that WES was performed only on the proband sample. This single-sample approach restricts our ability to fully assess the inheritance pattern and the broader genetic context of the mutation. To address this limitation, future research should include family-based sequencing to better understand how the mutation is inherited and its potential interaction with other genetic factors, providing a more comprehensive view of *ASH1L*-related disorders. Future studies could leverage multi-omics approaches to explore the transcriptional, proteomic, and epigenetic alterations resulting from *ASH1L* loss-of-function mutations, particularly nonsense variants. Transcriptomic analyses could provide insights into specific gene expression disruptions linked to *ASH1L* mutations, while proteomic studies might elucidate downstream signaling pathways that are dysregulated. However, there are significant methodological differences between the construction, tissue sampling, RNA preparation, and analysis of the two models. Moreover, the use of patient-derived induced pluripotent stem cells (iPSCs) and neuronal differentiation models could enable in-depth functional studies, helping to identify molecular targets for potential therapeutic interventions. Additionally, to understand how *ASH1L* haploinsufficiency affects cognitive and behavioral phenotypes, it would help to do long-term studies on groups of people and use brain imaging to see how specific brain areas change in structure and function over time.

In conclusion, the identification of the novel *ASH1L* p.Lys827* nonsense variant contributes significant insights to the complex genetic landscape of ID and *ASH1L*-related neurodevelopmental disorders. By broadening the clinical spectrum associated with *ASH1L* mutations, this study provides a foundation for genotype–phenotype correlations and emphasizes the necessity for comprehensive genetic and functional analyses in mild ID cases. Future research should continue to investigate the specific pathways affected by *ASH1L* mutations and explore potential targeted therapeutic interventions that address the potential epigenetic dysregulation in ASH1L-related disorders.

## Data Availability

The raw data supporting the conclusions of this article will be made available by the authors, without undue reservation.

## Footnotes

^1^ https://www.hgmd.cf.ac.uk/

^2^ https://www.uniprot.org/

^3^ https://golgi.sandbox.google.com/about

^4^ https://pymol.org/

^5^ https://string-db.org/

^6^ https://gtexportal.org/home/gene/*ASH1L*
